# Implications of Epigenetic Variability within a Cell Population for “Cell Type” Classification

**DOI:** 10.3389/fnbeh.2015.00342

**Published:** 2015-12-16

**Authors:** Inna Tabansky, Joel N. H. Stern, Donald W. Pfaff

**Affiliations:** ^1^Laboratory of Neurobiology and Behavior, The Rockefeller UniversityNew York, NY, USA; ^2^Departments of Neurology and Science Education, Hofstra North Shore-LIJ School of MedicineHempstead, NY, USA; ^3^Department of Autoimmunity, The Feinstein Institute for Medical Research, North Shore-LIJ Health SystemManhasset, NY, USA

**Keywords:** single cell analysis, cell type classification, variability in gene expression, *in vitro* culture, functional equivalence

## Abstract

Here, we propose a new approach to defining nerve “cell types” in reaction to recent advances in single cell analysis. Among cells previously thought to be equivalent, considerable differences in global gene expression and biased tendencies among differing developmental fates have been demonstrated within multiple lineages. The model of classifying cells into distinct types thus has to be revised to account for this intrinsic variability. A “cell type” could be a group of cells that possess similar, but not necessarily identical properties, variable within a spectrum of epigenetic adjustments that permit its developmental path toward a specific function to be achieved. Thus, the definition of a cell type is becoming more similar to the definition of a species: sharing essential properties with other members of its group, but permitting a certain amount of deviation in aspects that do not seriously impact function. This approach accommodates, even embraces the spectrum of natural variation found in various cell populations and consequently avoids the fallacy of false equivalence. For example, developing neurons will react to their microenvironments with epigenetic changes resulting in slight changes in gene expression and morphology. Addressing the new questions implied here will have significant implications for developmental neurobiology.

## Determination of Cell Types

Fundamental to modern cell biology is the idea of a cell type: a group of cells that shares similar properties and performs certain biological functions. During the past several decades, this basic idea has had to be adapted to multiple technological advances that challenged the way we identify and classify cells. We have now observed both variability in gene expression and functional differences in cells that could be considered the same cell type (Sheng and Greenberg, 1990; Rossi et al., 2005; Beerman et al., 2010; Blanpain and Fuchs, 2014; Marder et al., 2015). Understanding cell type classification in the context of these new technologies is a particular challenge for fields that study complex organs with many different cell types, such as neuroscience and immunology. Both disciplines face the daunting task of having to classify cells that may be similar in appearance and that alter gene expression patterns in the course of their normal function. Here, we address the evolution of the concept of cell type throughout history, the impact of new technologies, and how this concept might have to evolve in the future.

The concert of cell type continues to evolve, and in the nervous system, initial investigations of cell types, such as the pioneering work of Santiago Ramon y Cajal, relied both on morphology and location within the body (the brain and the gut). Due to the large variability of morphology found in distinct neuronal subtypes, it was possible to define many neurons such as pyramidal cells within the cortex and the interstitial cells of Cajal, the pace makers of the gut (Ramon y Cajal, 1909). Thus, initially, if a cell was located in a particular region of the brain and it possessed a certain appearance, it was classified as a particular type of neuron.

However, using morphology as the main designator of a neural cell type can cause a problem; not all neurons have a distinctive morphology. For example, simple, bipolar neurons are found in many regions of the central nervous system (CNS), but there has been no way to tell, from morphology alone, whether their biological functions are similar to, or significantly different from each other. The question then becomes; how do you really know whether two cells are the same “type”? If appearance is not the answer, what is?

## Markers to the Rescue?

Because the function of any cell is so dependent on its biochemistry, molecular characterization of cell types appears to be the next logical step. With the advent of revolutions in molecular biological methodology, it has become possible to characterize cell types further based on expression of “marker” genes, generally connected with their function. This idea has been enormously useful in neuroscience, particularly when discussing the signaling molecules that endow neuronal cells with their unique properties. For instance, a dopaminergic neuron must, by definition produce the enzymes necessary for making dopamine, and orexin neurons must produce orexin. By using marker genes, visualization of the cell gives clues to its function. Such a feat is impossible using even the most beautiful Golgi stain. Thus, the signature molecules of a particular neuronal cell type provide a more sophisticated route to cell type classification.

However, a cell type generally has multiple genes that are crucially necessary for function, and thus, the use of markers also presents a complicated problem with regard to the interpretation of cell type. For instance, orexin knockout mice (Chemelli et al., 1999) possess lacZ and neomycin resistance cassettes inactivating the orexin gene. Thus, instead of producing orexin, they produce β-galactosidase, an enzyme of bacterial origin that is capable of hydrolyzing sugars and turning certain colorless substrates a bright blue color. Despite the fact that the neurons in this mouse produce β-galactosidase and not orexin, they are still classified as “orexin” neurons, not β-galactosidase producing neurons. While on the surface, this point may seem obvious, it reveals that we actually define cell type by function, developmental history, and location, not gene expression by itself—even when the expression of a particular gene is required for a cell to perform its function. The fact that these neurons transcribe the genetic locus that used to encode orexin is their defining feature, regardless of the fact that in the mutated cells, this locus now encodes a completely different protein. Thus, the expression of a particular marker cannot by itself define a cell type, as in certain circumstances (e.g., mutations) a cell can still be a particular “type” without expressing the molecule that is crucial for function.

## Epigenetic Modifications—Crucial Determinants of Gene Expression

Since both morphology and marker expression by themselves are insufficient to define a cell type, how can such a determination be made? A strong argument could be made for epigenetic modifications, as they are determined by developmental history and cell origins, and clearly are necessary to regulate gene expression (Bird, 2007). Several types of epigenetic modifications exist, and major efforts are now being undertaken to understand the implications for gene regulation (Benveniste et al., 2014; Maze et al., 2014a,b).

For instance, DNA methylation has been thought until recently to be indelible due to the complexity of the chemical bond, until evidence for active DNA demethylation (Oswald et al., 2000; Hajkova et al., 2002), and subsequently, active DNA demethylases was discovered (Hackett et al., 2013; Moen et al., 2015). Analysis of DNA methylation in individual cells (bisulfite sequencing) shows considerable variability within cell populations, even in genetically identical individuals (Fraga et al., 2005; Martino et al., 2013). Based on current data, this variability is unlikely to decrease as technological progress is makes the technique more precise, as it appears to reflect intrinsic biological variability (Mo et al., 2015). Even in more recent large-scale methylation analyses of multiple loci conducted on individual pluripotent cells with single-molecule resolution that are able to retrospectively group cells according to methylation patterns, there is considerable variability in the amount of methylation molecule-to-molecule (and certainly CpG to CpG), necessitating hierarchical clustering and other statistical approaches to derive patterns from the data (Wernig et al., 2007; Meissner et al., 2008; Chavez et al., 2010). Thus, while a “signature” DNA methylation profile for a cell type may be derived, it may not always be applicable to individual cells, due to what currently appears to be intrinsic variability.

The above observations may of course change, because—as all biologists recognize—distinguishing “genuine” biological variability, which reflects real differences in gene expression or modification, from technical variability, which is a result of sampling error while running an experiment, presents a considerable problem. This is especially true in techniques where cells must be destroyed to make the analysis, precluding *post hoc* functional correlation. The DNA for bisulfite sequencing is usually isolated from populations and cells are destroyed during the analysis. Thus, reconstruction of their functional history is a challenge, though single cell approaches are currently in development (Farlik et al., 2015). A further technical complication is the inability of bisulfite sequencing and enzymatic approaches to distinguish between 5-methylcytocine (5-mC), and 5-hydroxymethylcytosine (5-hmC), the intermediate DNA modification found during active demethylation, and has been implicated in several biologically important processes (Nestor et al., 2010; Freudenberg et al., 2012; Teif et al., 2014). Due to the distinctive biological functions of 5-mC and 5-hmC, the interpretations of the results of bisulfite sequencing are therefore complicated, as without knowing the exact nature of the chemical DNA modification, functional consequences are not necessarily predictable from the data. Even more vexingly, even if gene expression can be tied directly to the prevalence of signal from bisulfite sequencing, it would be unclear whether 5-mC or 5-hmC is responsible for causing that change.

DNA methylation is not the only epigenetic modification the implications of which for gene expression and function are not fully understood. While certain types of histone modifications are thought to serve as hallmarks of a particular developmental state and/or type—for instance, the so-called “bi-valent” modification are a sign of a progenitor or pluripotent cell (Bernstein et al., 2006)—but the relationship between histone modification and gene expression is not straightforward. Additionally, most analyses of histone modifications address aggregate cell populations, rather than individual cells. For genome-wide analysis of histone modifications, the current state of the art is 10^3^ cells, and Western blots require large numbers of cells (Brind’Amour et al., 2015). Thus, while analysis of histone modifications can be used to bolster an argument that a particular cell population exhibits a histone signature of a particular “type”, making such a determination on the basis of histone modifications alone is currently impractical. Like DNA methylation, it is possible to classify cell populations based on chromatin states (Ernst et al., 2011), but due to the current impossibility of single-cell analysis, the reverse (classifying individual cells into types based on chromatin state) is technologically not feasible. Thus, it may be a little while before histone modifications are used as part of the definition of cell type.

## Using RNAseq to Determine Cell Type

Using gene expression as part of cell type classification is logical both because it at least partially reflects the proteins found in—and thus the biochemistry—of a cell, and also as it is the presumed direct output of epigenetic modification. Current technology focuses primarily on RNA expression, allowing relative quantification of RNA on a single-cell level (Tang et al., 2010a; Islam et al., 2014; Jaitin et al., 2014; Pollen et al., 2014; Saliba et al., 2014; Treutlein et al., 2014; Wu et al., 2014; Zeisel et al., 2015). The power of single-cell RNAseq relies at least in part on statistical methods of clustering individual cells according to their gene expression patterns (Tsafrir et al., 2005; Hebenstreit, 2012; Jaitin et al., 2014; Saliba et al., 2014; Treutlein et al., 2014; Wu et al., 2014; Satija et al., 2015). Hierarchical clustering approaches allow the classification of single cells within a tissue into distinct cell types, without a-priori assumptions about expression of markers, etc (Tsafrir et al., 2005; Jaitin et al., 2014; Pollen et al., 2014; Treutlein et al., 2014). Such an approach would likely help identify the “natural” cell types within populations, and may reveal differences between cell type classification and identifying markers.

For neuronal populations, in particular single cell RNAseq appears to reveal an unexpected molecular complexity (Lodato et al., 2011; Molyneaux et al., 2014; Lodato et al., 2015; Macosko et al., 2015). How this apparent inconsistency in gene expression within a cell type contributes to the protein content or function of cells remains to be seen. Because cells are destroyed for RNAseq, it is impossible to follow them over time. It is possible that the variation of gene expression is due to environmental factors, or alternately, that it is “permissible” within a given cell because post-transcriptional regulation ensures a standard complement of proteins and therefore, standardized function. Alternately, it is also possible that such variability is an intrinsic feature of cells, with multiple cell types existing on a continuous spectrum that overlaps. More sophisticated technologies are needed to address this question.

In addition to uncertainty with regard to post-transcriptional regulation, it is currently impossible to combine RNAseq with techniques to detect differences in rates of translation, rates of protein degradation, and post-translational modifications in cell types. While gene expression may indicate that a protein is likely present, most protein are active only if they are within an appropriate compartment in a cell, and it is not currently possible to elucidate protein localization in cells used for RNAseq, though high throughput techniques to determine protein localization are currently being developed (Briley et al., 2015). Other techniques, such as retrograde tracing with viruses also need to be combined with RNAseq in order to narrow the search for gene expression to neurons projecting to specific brain regions (Ekstrand et al., 2014).

An even more daunting challenge for researchers using single-cell RNAseq for cell type identification is the question of how gene expression correlates with function, especially given the need to define cell types increasingly by function (Kepecs and Fishell, 2014). Especially in the context of studies indicating that cell molecular diversity is greater than the current known functional diversity, this is a considerable challenge. RNAseq produces huge quantities of data, and the effect of every gene expression change on functionality, especially in the context of the nervous system, cannot reasonably be tested with current technology. Additional problems arise when a researcher is using RNAseq to prove that a cell type *in vitro* is sufficiently similar to an *in vivo* cell type to perform functional tests, and even more so when the *in vivo* cell type is not readily available, such as in the case of human neuronal cell types derived from human pluripotent stem cells. Thus, RNAseq, and especially single-cell RNAseq are excellent tools for exploring the limits of what it means to be a cell type, due to their capacity to detect large numbers of molecular variations within a cell. However, data from these analyses must be interpreted with caution, especially in light of a paucity of information on post-transcriptional and post-translational regulation.

## Multimodal Gene Expression in Cell Types Outside the Nervous System

While the primary focus of this discussion is on the nervous system, it is important to note that multiple cell types that interface with the nervous system show similar multimodal gene expression and functional variability. Especially crucial for a system that interfaces intensively with the CNS, different immune cells can have differential expression of protein levels, phenotypic output and gene expression resulting in functional variation (Bengtsson et al., 2005; Raj and van Oudenaarden, 2009; Feinerman et al., 2010; Kalisky et al., 2011; Shalek et al., 2013). For example, dendritic cells (DCs) the primary antigen presenting cells and immune modulators—may not all be the same simply because they express the same cellular markers. When subpopulations of immune cells are sorted based on surface antigens, we assume that the surface antigens are reflections of the biological function of the cell. For instance, if we want to obtain B cells we will sort for B lymphocyte antigens positive (CD20 or CD19) cells. Notably, CD19 is also expressed on follicular DCs. Even beyond the caveat of non-specificity, these so-called “marker genes” are not all equally crucial for the function of the cell type that they define.

In some cases, knocking out the marker of an immune cell population does very little to alter the functionality of this population, while in others, all functionality is lost. For instance, CD11b (Mac-1) knockout mice, while they have slight aberrations in the function of monocytes (CD11b^hi^), still possess that cell type which is mostly able to fulfill their function (Lu et al., 1997). Despite the relative normalcy of monocytes in these knockouts, CD11b appears to be crucial for the function of neutrophils, as this cell population exhibits major defects in knockout mice. In a more extreme case, when CD4 T cell receptor is knocked out in mice, these animals lose almost the entire population CD4^+^ T cells (Rahemtulla et al., 1991). With the advent of single cell RNA sequencing, it is likely that both the molecular signatures and the molecular diversity of various immune cells will be identified. This will present additional challenges with regard to classifying populations of immune cells and their functions, and will have significant implications for the study and treatment of neurodegenerative diseases and infections.

Because immune cells have both active and inactive states, and also multiple states of maturation, there is additional complexity in interpreting gene expression data from these cells. For instance, in order to investigate the heterogeneity of immune cells, DCs were derived from bone marrow and stimulated with lipopolysaccharide (Shalek et al., 2013). The researchers found differences in RNA splicing patterns and levels, which were used to show cell heterogeneity (Shalek et al., 2013). Additionally, immune cell that had similar bimodal expression of genes may be closely related, reflecting different cell maturity states. Thus, cells that are technically the same “cell type” can have very different gene expression patterns, depending on their functional state.

This property of multiple gene expression signatures within the same cell type appears to be wide spread. For instance, in stem cell compartments in epithelial tissue, gene expression and functionality can be bimodal: the cells can display altered properties depending on whether they are activated or quiescent. Despite these differences, epithelial stem cells share the property of “stemness”, and their behavior is determined by their niche (Blanpain and Fuchs, 2014). Even in the earliest cells resulting from the first cell division of the zygote, functional differences in their properties may be observed (Tabansky et al., 2013). Thus, while a group of cells with very similar functional properties (i.e., different types of neurons) may be considered different cell types, cells with very distinct molecular profiles (i.e., epithelial stem cells) may be considered to be the same cell type.

The functional and molecular properties of cells can also shift with age, as found in the hematopoetic system. With age the population of hematopoetic progenitors shifts produces increased numbers of myeloid cells, at the expense of lymphoid cells (Rossi et al., 2005; Beerman et al., 2010). While hematopoetic cells remain hematopeotic cells, their function and gene expression patterns shift considerably, potentially resulting in a decrease in immune function and increased rates of leukemia (Rossi et al., 2005; Beerman et al., 2010). Thus, a cell type can show a large amount of variation in gene expression and function, while remaining the same cell type.

In all the examples described above, the cell types have a function whose outcome is readily measurable. This may be because a precise functional definition makes cell type classification more clear, making it easier to determine whether the molecular variability necessitates classifying certain cells as multiple cell types. In contrast, the nervous system comprises a large number of closely interrelated cell types that all function together, making functional definition of cell types a daunting challenge. Adding to the complexity is the fact that the final output of the system (behavior and physiological functions) requires so many different components that it is nearly impossible to deconvolute what, exactly, each cell type contributes to the system without extensive work. However, if we take a lesson from other cell types, it is likely that individual neuronal cell types are not standardized in their functional output or gene expression. In fact, there are many potential sources that must be considered when analyzing variation of gene expression in neurons, including circadian cycles (Ko et al., 2001).

Eve Marder (Grashow et al., 2010; Tang et al., 2010b) has already faced the questions of neurophysiological similarity in neurons performing different functional roles level. In the context of system performance by a complex neural circuit, obsession with fixed averages, highly tuned, often would not be appropriate: by avoiding a focus on each individual component of a system, and being sensitive to relations between components, “permitted” variability might be understood. In particular, compensation in the value of one parameter to variations in another will be important to study. For example, using four neurons or types recorded from the crab stomatogastric ganglion, each of which showed marked variability for each intrinsic property considered, (Grashow et al., 2010) were able to report that under some conditions similar circuit performance was achieved despite marked variability within “neuronal types”. Notably, the stomatogastric ganglion functions well in the presence of a variety of rhythmic activities (Bucher et al., 2006) perhaps, precisely, because these temporal patterns show “bidirectional interactions and coordination” (Marder et al., 2015).

This approach to variability within neuronal activities is rare because more complex neuronal systems have not had individual neuronal “types” so well characterized. However, due to their unique shape and distribution of functions, pyramidal neurons in the primate cerebral cortex are similarly amenable to analysis of variation in electrophysiology, numbers of dendritic spines and architecture of dendritic trees. Importantly for researchers working on common model organisms, the structure of pyramidal neurons in the cortex is different in different species of animals, perhaps reflecting differences in function (Elston, 2007). In even more striking results, individual neocortical neurons appear to change in neurophysiological properties and dendritic arborization throughout the lifetime of the animal (reviewed in, Jacobs and Scheibel, 2002; Spruston, 2008; Elston and Fujita, 2014), thereby undermining the concept that cortical “cell types” have are functionaly equivalent throughout the lifetime of the animal or in different locations a particular brain region.

On a molecular level, the gene expression patterns of the neurons in the cerebral cortex are increasingly being characterized on a single-cell level (Lodato et al., 2011, 2015; Molyneaux et al., 2014). The results of these analyses indicate that the molecular features of the neurons are integral to the types of projections that these cells make, and also that the molecular diversity of cell types far exceeds the diversity of cell types observed by electrophysiology and morphology. Thus, at least in the cerebral cortex, neurons that are anatomically analogous (appear to have similar function, but perhaps different developmental origins) may in fact be homologous in terms of gene expression (do not perform the same function on a molecular level, but appear similar). This is particularly important as slight variations in the types of neurons present, especially during development, could alter the local micro circuitry, (Lodato et al., 2011, 2015; Molyneaux et al., 2014) with potential consequences for behavior and mental health.

The variability of gene expression and physiological properties based on functional state is an issue that needs to be addressed when definitions of cell types—particularly neural cell types—are considered.

## Arguing Similarities Between Cell Type-Logic and Species-Logic

Given the biological complexities of classifying cells, a decision must be made about what sorts of criteria can be reasonably used to distinguish cell types. In many ways the idea of a cell type is very similar to the idea of a species: it exists both as a biological entity and a concept that allows convenient parsing of individuals into simple groups. In evolutionary biology, multiple definitions of species exist, even though the most commonly used is the biological species concept (Futuyma, 1998). As a result, species as defined in one concept will not necessarily be species as defined by another. Notably, after molecular analysis of the genetics of species became possible, new definitions of species were proposed. One of these definitions, the genealogical species concept, relies on the presence of consistently different alleles at the same genetic loci in two different populations of animals (a monophyletic gene tree; Futuyma, 1998). Thus, populations are grouped by consistent genetic differences (with some caveats), similar to hierarchical clustering used to group populations of cells into cell types by RNA-seq (Futuyma, 1998). Thus, both cell type classification and species classification can vary dependent on the complexity of information used to make the distinction; but using more complex information is not always more accurate.

The genealogical species concept, which is based on a variety of genetic information sometimes prevents appropriate classification. Using the genealogical species concept sometimes divides the biological unit constituting a group of interbreeding individuals into multiple subgroups, and occasionally clusters two distinct biological units together (Futuyma, 1998). In this way, the challenges faced by the genealogical species concept are no different from challenges faced by hierarchical clustering: how do you determine whether the “cell type” identified by RNAseq is a true biological unit? For instance, if one cell expresses c-Fos and another does not, they are still likely to be the same cell type displaying different functional states (Sheng and Greenberg, 1990). In contrast, a dopaminergic neuron that does not express appreciable levels of tyrosine hydroxylase (or at least activated the tyrosine hydroxylase gene, if it is mutated) is not a dopaminergic neuron, as this enzyme is crucial for dopamine synthesis. Thus, the question of whether two individuals can be classified into two groups based on any given difference is determined by the impact of this difference on the function of the individual, both in the cases of cells and in whole organisms.

Despite the similarities of the species and cell type concepts, there are clear differences between the two forms of classification. While for a species, the lineage is generally presumed to be defined by the ability of its members to share genes as a community, somatic cells in an organism do not undergo sexual reproduction. In contrast, the most important definition of a cell is the ability to perform its biological function within the context of an organism. Therefore, any definition of a “cell type” must be functionally based.

**Figure 1:**
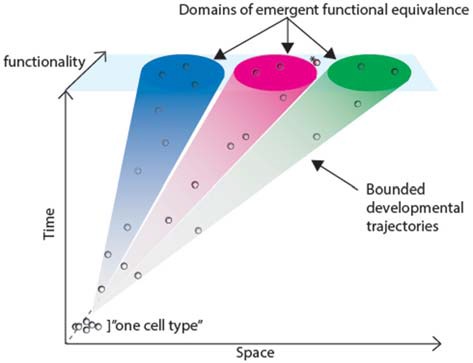
**Changes in functionality and gene expression of cells in space and time.** Starting at E0, cells follow trajectories from an undifferentiated state to functionally mature states, trajectories through both space and time, gradually accumulating steadily more divergent developmental histories. The colored cones represent emergence into functionally equivalent domains. Some cells may migrate slower than others, but all must end up in the right area of the body to perform their normal physiological function. The cells acquire the properties of the mature cell type as they move up the idealized developmenta‘l trajectory. Once they reach the “peak” of the trajectory, the cells are permitted to have slight differences, as long as they maintain their function. In some cases, accumulated differences may be enough for a cell to fall out of the functional domain (asterisk).

*Cell “phenotype” instead of cell type?* If we were to take a brief lesson from evolutionary biology, two cells should be classified as the same “type” *if and only if they do not have any gene expression/epigenetic differences that interfere with their ability to contribute in a similar manner to the function of the organ in which they are found*. Such an approach replaces the idea of a cell “type” with a cell “phenotype”, and incorporates the natural functional variation already found in many kinds of cells, including neurons (Rahemtulla et al., 1991; Lu et al., 1997; Rossi et al., 2005; Beerman et al., 2010; Blanpain and Fuchs, 2014; Marder et al., 2015). In practice, hierarchical classification of distinct cells by gene expression is a first attempt to identify the natural cell type; but like the genealogical species concept, functionality is the ultimate readout. However, the function of each cell within a “phenotype” could be allowed to vary, to some degree (Figure 1). In order to truly determine which cell type a cell belongs to, it is therefore necessary to acquire a deep understanding in the role that genetic variability plays in the function of that cell type.

## Shaping Cell Type Identity Through Time

Unlike animals—who cannot change their genetic code or their major morphological characteristics—cells are able to change gene expression patterns and epigenetic modifications, moment-to-moment. In fact, expression of genes necessary for function is known to oscillate or alter in some cell types as a result of aging, cell division, circadian rhythms or other factors (Figure 1; Sheng and Greenberg, 1990; Rossi et al., 2005; Beerman et al., 2010; Blanpain and Fuchs, 2014; Marder et al., 2015). Thus, any definition of a cell type based on molecular data such as RNAseq would have to account for that variability.

**Figure 2:**
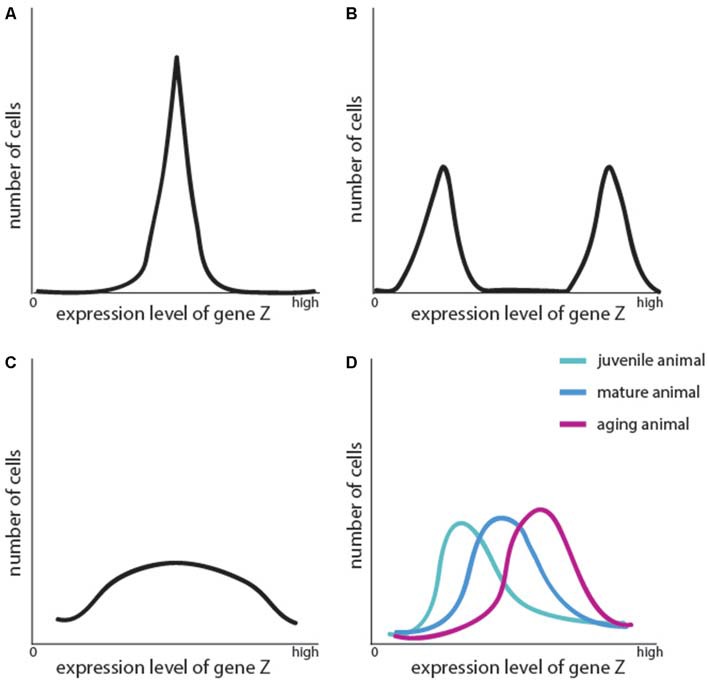
**Frequency distribution of cells according to the level of expression of an arbitrarily chosen gene “Z”.** Here are four abstract examples of such patterns. **(A)** All cells express gene Z at the same level, forming a coherent peak, as would usually be expected to occur in cells of the same cell type. **(B)** A seemingly homogenous population has two peaks of expression for gene Z (some cells express it strongly, and some not at all). This could be taken to mean that these are two distinct cell types, but first, two considerations must be addressed: whether gene Z is functionally relevant and whether gene Z has a transient, oscillating expression pattern that reflects diverse functional states. Examples of the latter would be immediate early genes in neurons, or genes responsible for mitosis. **(C)** A population has no distinct pattern of expression of gene Z. This could be because there are in fact numerous subpopulations present within this population, or because gene Z expression oscillates very slowly between expression and degradation. **(D)** The expression of gene Z shifts with age, altering the function of the cells; but due to the slow progression, the cell type could be argued to remain the same.

The distributions of variation of gene expression would be expected to be variable depending on whether particular levels of that gene are necessary, or whether it varies based on the functional state of the cell. If a population has a variable expression of a gene necessary for function, then a certain distribution can be predicted for the expression of the gene; the distribution of the levels of the gene in the cells would form an extremely broad peak, potentially even making it look as though the analysis was flawed (Figure 2). If, in contrast, the differences are a result of two separate cell populations being mistaken for one cell population, the curve could have two peaks. This would provide a mathematical clue as to the behavior of the gene in the population, but the expression of the gene over time and in different functional contexts would need to be examined in order to conclude that it does not represent two distinct functional states. For genes whose pattern of expression alters based on aging, it is necessary to assess expression level at multiple time points (Figure 2). Thus, not only the curves of patterns of gene expression, but also functional implications of the observed distribution in gene expression need to be carefully considered in these analyses.

For certain transient cell populations that do not persist into adulthood, such as radial glia, a cell type would have to be considered not only in terms of gene expression, but also in terms of time. If a cell with the complete gene expression pattern and pattern of epigenetic modifications of radial glia were located, for instance, in an adult liver, with no positional cues to allow proper neuron formation, it could be argued that this radial glial cell is merely a “radial glial-like” cell. However, if there would be invented a technical means of introducing such a cell into the embryonic CNS, where it is able to fulfill its function, such a cell would become a radial glial cell. To continue the analogy between cell type and species, removing a cell type from its normal environment is much like removing an animal from its ecological niche. Neither the cell nor the animal would be able to function normally outside the niche, and it would be difficult to predict whether they would be able to survive and function in their normal environment once they are no longer in that environment. Thus, an integral part of any classification of cell type would require the consideration of the location and normal developmental timing of that cell.

We therefore call for shift of emphasis from static concepts to dynamic concepts that allow for cell-to-cell variation, while focusing on trajectories that lead cells to develop functional adequacy within the brain (Figure 1).

## Methodological Challenge Posed by this Logic

Even taking into account the advances in sequencing techniques and in epigenetic chemistry during the last two or three years, it must be stated that we currently have only limited tools to test the concepts presented here. Fundamentally, we have to be able to assess the *transcriptional activities and functionality* of living cells at more than one point in time. That is, we must follow the flow of gene expression through time, to know “where the cell has been” and where it is going. It may eventually be able to use intracellular lasers for this purpose (Schubert et al., 2015; Humar and Yun, 2015). At the moment, the only way to do so is through expression of reporter genes, fusion proteins, and site specific recombinases systems. Minimally invasive methods of characterizing expression of large numbers of genes through time would be desirable for analyzing the relationship between gene expression dynamics and cell function.

## Implications for *in vitro* Culture Systems

If cellular function depends in part on location, how do we reconcile it with *in vitro* culture, particularly the burgeoning field of stem cell modeling of diseases and development?

One of the strengths of the stem cell field from its inception, has been an emphasis on functional outcomes (Nagy et al., 1990, 1993; Kang et al., 2009; Blanpain and Fuchs, 2014). No matter how deeply the transcriptome and epigenome are studied, it is philosophically and empirically impossible to argue that a cell type existing in a dish is the same as the cell types found in the body.

The *in vitro* environment by its nature requires that cells adapt and alter their gene expression in order to survive. *In vitro* oxygen concentrations are much higher (Brewer and Cotman, 1989; Morrison et al., 2000; Studer et al., 2000), pH is more variable, and the extracellular matrix and supporting cells are quite different (Eagle, 1971; Coutu and Schroeder, 2013). Much effort has been expended to make culture of nervous system cells as close to physiological as possible, while still allowing analysis. These strategies include, but are not limited to, organotypic culture, addition of supportive cell types, 3-dimensional gel culture, and minimizing evaporation to reduce tonic stress (Whatley et al., 1981; Gähwiler, 1981; Huettner and Baughman, 1986; Bolz et al., 1990; Stoppini et al., 1991; O’Connor et al., 2001; Hayman et al., 2005; Gelain et al., 2006; Cullen et al., 2007; Ylä-Outinen et al., 2014). Experimentally, all these methods are extremely useful for construction of better models of diseases and biological conditions. However, it remains true that the *in vitro* environment is quite different from the *in vivo* environment, and deviations in cell function are to be expected.

Based on the theoretical definition of cell phenotype advanced in the present discussion, *in vitro* cultured cells can never be considered the same cell type as cells *in vivo*; but they can still serve as functional models. This caveat would remain even if gene expression is identical, as it would still be impossible to confirm that all possible sources of decreased functionality have been tested, and to eliminate the hypothesis that there are biologically significant differences between the two cells. Paradoxically, a refusal to qualify the *in vitro* cell types as identical to “natural,” *in vivo* cell types would make them more useful. Assuming that the *in vitro* cells are able to functionally model at least some of the properties of their biological counterparts, multiple systems for *in vitro* modeling may be more useful than attempts to come up with cells that perfectly models the “natural” cell types.

Recognizing and accepting the limitations of *in vitro* models as part of the process of exploration, as opposed to a flaw is especially important considering how much time is spent looking for superior models of diseases and biological processes, especially in light of the differences between human and mouse in cellular function (Cheng et al., 2014; Pope et al., 2014; Stergachis et al., 2014; Yue et al., 2014). An additional incentive is the failure of certain mouse genetic models to replicate human disease, necessitating the development of human cell-based models (Tiscornia et al., 2011). In the context of the natural and artificial variability of cell populations, it is increasingly important to imitate physiological conditions as closely as possible (Gähwiler, 1981; Whatley et al., 1981; Huettner and Baughman, 1986; Bolz et al., 1990; Stoppini et al., 1991; O’Connor et al., 2001; Hayman et al., 2005; Gelain et al., 2006; Cullen et al., 2007; Beerman et al., 2010; Ylä-Outinen et al., 2014). However, creating a diversity of models of various cell types is in itself a laudable experimental goal. This is especially true given the natural variability of *in vivo* cell populations (Sheng and Greenberg, 1990; Rossi et al., 2005; Beerman et al., 2010; Blanpain and Fuchs, 2014; Marder et al., 2015), and it is likely to become an important consideration in drug development: testing a drug on multiple cell-based models and observing similar results would perhaps raise the bar on the number of drugs admitted into clinical trials, but it may also reveal mechanistic failures of the drugs before they enter clinical trials. Thus, designing multiple *in vitro* models of disease, each of which mimics at least some *in vivo* aspect may be a more rigorous form of screening than aiming for one ideal model.

*Thus*, the generation of a specific nerve cell type depends, at least in part, on epigenetic chemistries, leading to temporally modulated changes in gene expression, leading, in turn, to the biologically appropriate neuronal function. Our concepts of how these trajectories are regulated during development will likely continue to evolve as whole-cell analyses of multiple cell processes continue to advance technologically. In addition to analyses of all the proteins found within a cell (proteomics), analyses of active kinases or carbohydrate groups attached to proteins will continue to provide more subtle gradation of variation within cells, likely leading to an explosion of definitions of cell state (Shi et al., 2012; Cheng et al., 2014; Pope et al., 2014; Stergachis et al., 2014; Yue et al., 2014; Briley et al., 2015; Farlik et al., 2015). It is important for us to remember that a cell type is by itself an artificial distinction, created for the convenience of biologists, much in the same way as the concept of a species is created for us to distinguish between animals. As finer and finer gradations of cellular state become available, it may be tempting to classify each transient event as an indication of permanent, fundamental difference between two cell populations. As ways to measure minute differences between cells become more and more sensitive, a continuous engagement with the concept of cell type will become of paramount importance so that cells can be appropriately classified and studied.

## Summary

We have re-examined the problem of defining nerve “cell types”, and suggested an approach to nerve cell equivalence classes that goes beyond the conventional, imperfect definition. One of the most fundamental concepts in cell and developmental biology is that of the “cell type”: the claim that a certain group of cells shares identical structure and function. However, this “cell type” concept has sometimes fallen prey to a fallacy of false equivalence. That is, this conventional model of cell equivalence has been recently undermined by advances in single-cell gene expression, lineage tracing, and genome sequencing. Among cells thought to be equivalent, biased tendencies among differing developmental fates have been demonstrated within multiple lineages in the body. Single-cell RNA sequencing, within a seemingly homogenous cell population, now uncovers considerable differences in global gene expression patterns. These observations show that the current model of classifying cells into distinct types must be revised to account for the intrinsic variability that exists within apparently identical cells in a population. We have suggested that a “cell type” could now be defined as a group of cells that possess similar, though not identical, functional properties, variable within a spectrum of epigenetic adjustments that, most importantly, permit its biologically adaptive developmental path, resulting in a functionally equivalent class of cells, to be achieved.

Essential properties for a cell type designation would likely include not only morphology, but also expression of certain “core” genes coupled with specific epigenetic modifications. Crucially, the ultimate criterion for equivalence would depend simply on whether this cell is able to fulfill its function within the organism. Thus, “emergent functional equivalence”. This approach accommodates avoids the fallacy of false equivalence to which some previous cell biological approaches have fallen prey. It could lead to incisive investigations into which properties of a nerve cell actually confer functionality. Overall, we call for a shift of emphasis from static concepts—those claiming absolute cell equivalence *ab initio*—to dynamic concepts that allow for cell-to-cell variation, while focusing on trajectories that lead cells to develop functional adequacy within the brain. For example, developing neurons will react to their microenvironments with epigenetic changes resulting in slight changes in gene expression and morphology, without affecting their intrinsic functional capabilities.

Our thinking implies new methodological challenges even as it offers definitional clarity for the cell biology of neurons during development.

## Funding

IT was funded by the Rockefeller University’s Bristol Myers Squibb Postdoctoral Fellowship and by National Institute of Child Health and Human Development F32 HD081835. JNHS was supported by a grant from the HF Langbert Neuroimmunolgy Research Award from the Department of Neurology, North Shore-LIJ Health System and from the Cheryl Manne Research Fund for the Cure of MS.

## Conflict of Interest Statement

The authors declare that the research was conducted in the absence of any commercial or financial relationships that could be construed as a potential conflict of interest.
